# DNA word analysis based on the distribution of the distances between symmetric words

**DOI:** 10.1038/s41598-017-00646-2

**Published:** 2017-04-07

**Authors:** Ana H. M. P. Tavares, Armando J. Pinho, Raquel M. Silva, João M. O. S. Rodrigues, Carlos A. C. Bastos, Paulo J. S. G. Ferreira, Vera Afreixo

**Affiliations:** 1 Department of Mathematics & CIDMA, University of Aveiro, Aveiro, Portugal; 2 Department of Medical Sciences & iBiMED, University of Aveiro, Aveiro, Portugal; 3 Department of Electronics, Telecommunications and Informatics, University of Aveiro, Aveiro, Portugal; 4 IEETA, University of Aveiro, Aveiro, Portugal

## Abstract

We address the problem of discovering pairs of symmetric genomic words (i.e., words and the corresponding reversed complements) occurring at distances that are overrepresented. For this purpose, we developed new procedures to identify symmetric word pairs with uncommon empirical distance distribution and with clusters of overrepresented short distances. We speculate that patterns of overrepresentation of short distances between symmetric word pairs may allow the occurrence of non-standard DNA conformations, such as hairpin/cruciform structures. We focused on the human genome, and analysed both the complete genome as well as a version with known repetitive sequences masked out. We reported several well-defined features in the distributions of distances, which can be classified into three different profiles, showing enrichment in distinct distance ranges. We analysed in greater detail certain pairs of symmetric words of length seven, found by our procedure, characterised by the surprising fact that they occur at single distances more frequently than expected.

## Introduction

The similarity between the frequency of complementary nucleotides in a single strand of DNA is known as Chargaff’s second parity rule^1^. An extension to this parity rule suggests that, for each DNA strand, the proportion of an oligonucleotide (a sequence of adjacent nucleotides, also referred to as a genomic word) should be similar to that of its reversed complement, a property that has been studied both for prokaryotes and eukaryotes^2, 3^.

The origin of single strand symmetry is a topic of great interest, because it can contribute to the study of the origin and evolution of genomes. Currently, there is no single accepted justification for the intra-strand symmetry, although several hypotheses about its origin have been proposed^4^. It has been suggested that the occurrence of secondary DNA structures, such as stem-loops and cruciforms, is associated with the DNA symmetry phenomenon. Cruciforms are structures with four arms that can be formed at sites containing reversed complementary words. They are relevant in biological processes, including those of replication and transcription, recombination and translocation^5^. Because these structures are associated with genome instability, the determination of their occurrence in the human genome and the identification of the corresponding sequence motifs is of paramount importance, both in the context of disease development and evolutionary events^6, 7^.

Here, we address the distance distribution of symmetric word pairs and investigate the different distance profiles in the human genome. In particular, we develop a procedure to identify genomic words with patterns of overrepresented short distances (<1000 bp). Overrepresented distances are those that have observed frequency higher than the expected frequency predicted by an adequate model, in a statistically significant way. We suggest that patterns of overrepresentation of short distances between reversed complements may be related to the occurrence of cruciform structures, and we evaluate this hypothesis in the human genome. We study the distance distribution between reversed complements, in order to provide knowledge about the words that are strong candidates to the formation of cruciform structures in human DNA. Procedures based on inter-word distances have already been found useful to study genomic sequences, e.g., to detect CpG islands^8^ and to compare species^9^. The study addressed in this paper shows yet another use of inter-word distances and distance distributions, which may lead to a deeper understanding of intra-strand symmetry and its connection with secondary DNA structures.

## Materials and Methods

### Materials

We used the complete DNA sequences of the human genome, downloaded from the website of the National Center for Biotechnology Information (NCBI). We processed the available assembled chromosomes (GRCh38.p2) as separate sequences. All ambiguous or unsequenced nucleotides, i.e., all non-ACGT symbols, were considered sequence delimiters.

We also used pre-masked sequences^10^ available from the UCSC Genome Browser (http://genome.ucsc.edu) downloads page. These files contain the same GRCh38 assembly sequences, but with repeats reported by RepeatMasker^11^ and Tandem Repeats Finder^12^ masked by Ns.

To address the problem of possible assembly artefacts, we also used the whole-genome shotgun assembly (WGSA, to which we refer as “Celera”) of the human genome generated at Celera in December 2001^13^, and the May 2007 HuRef genome of J. Craig Venter, sequenced with capillary-based whole-genome shotgun technologies using the Applied Biosystems 3730xl DNA analyser, and de novo assembled with the Celera Assembler^14^, to which we refer as “HuRef”.

### Distance between symmetric word pairs

Consider the alphabet ${\mathscr{A}}=\{A,C,G,T\}$ and let *w* be a symbolic sequence (word) defined in ${{\mathscr{A}}}^{k}$, where *k* is the length of *w*. In this work, the pair composed by one word, *w*, and the corresponding reversed complement word, *w*′, is called a symmetric word pair. For example, (*AC*, *GT*) is a symmetric word pair.

We are interested in finding the distance between a given *w* and *w*′, with no *w* or *w*′ between them. As an example, consider *w* = *AC* and the sequence $\underline{AC}T\underline{AC}TCC\overline{GT}\underline{AC}TATA\overline{GT}C\overline{GT}$. In this example, there are three occurrences of the word *AC* (underlined), but only the 2nd and the 3rd occurrences are considered for the calculation of distances to their nearest reversed complements (overlined), since between the 1st and the 2nd occurrences of *w* there are no occurrences of *w*′. Distances are measured between the start positions of the words, so a distance *d* between reversed complements of length *k* implies that the words are separated by (*d* − *k*) intervening nucleotides. In this example, *d* = 5 for the first (*AC*, *GT*) pair and *d* = 6 for the second.

Distances *d* < *k* may only occur if a suffix of *w* matches a prefix of *w*′. On the other hand, *d* = *k* is impossible for words such as *CGCG*. To avoid this dependence on the specific composition of *w*, distances *d* ≤ *k* are not considered for analysis.

The distribution of the *distances of nearest reversed complements* (*DNRC*) is denoted as *f*_*w*,*w*′_. Note that *f*_*w*,*w*′_ may be different from *f*_*w*′,*w*_.

For a fixed word length, *k*, we are also interested in the overall DNRC distribution across all the symmetric word pairs. We define the *global DNRC distribution*, *f*_*k*_, as a weighted sum of the DNRC distributions of all symmetric word pairs with words of length *k*,

1 $${f}_{k}(d)=\sum _{w,w^{\prime} \in {{\mathscr{A}}}^{k}}\frac{{n}_{w,{w}^{^{\prime} }}}{n}{f}_{w,{w}^{^{\prime} }}(d),\,d > k,$$

where *n*_*w*,*w*′_ is the number of observations of nearest pairs of symmetric words (*w*, *w*′) of length *k*, and *n* is the total number of such distances. Only the analysed distances (*d* > *k*) are counted in *n* and *n*_*w*,*w*′_.

For generating the symmetric words, we used a simple algorithm that, for each position in the DNA sequence, *i*, and associated word of size *k*, *w*, searches for the first occurrence of *w*′. If *w* is found before *w*′, the algorithm skips to the next position *i*. For practical reasons, a maximum searching distance is specified by the user, allowing the program to maintain in memory a table with all possible words *w* and the corresponding number of occurrences at each distance.

In order to study the behaviour of the empirical global DNRC distributions of the human genome, *f*_*k*_, we carried out comparisons with the DNRC distributions obtained from nucleotide sequences generated by a *k*-order Markov process (random background). The expected global DNRC distribution under *k*-order Markov dependence, ${f}_{k}^{e}$, can be deduced using the transition probabilities and a state diagram that represents the progress made towards identifying *w* or *w*′ as each symbol is read from the sequence. The algorithm used to find this exact distribution^15^ is a special case of Fu’s procedure based on finite Markov chain embedding^16^.

#### Parameter assumptions

The stem and loop lengths of hairpin/cruciform structures seem to vary over a wide range. According to different authors, the stem length varies between 6 and 100 nucleotides, while loop lengths may range from 0 to 2000 nucleotides^6, 17, 18^.

Since this study intends to characterise the short distances between symmetric words, but avoiding the direct word dependencies, a range of distances from (*k* + 1) to 1000 was considered for computing all the DNRC distributions. Taking into account computational limitations and the possible stem length of cruciform structures, the histograms of the DNRC were computed for all symmetric word pairs of lengths up to seven, for all human chromosomes. For each of these sequences, a global DNRC distribution, comprising all symmetric word pairs of the same length, was also determined.

#### Chromosome homogeneity

To assess the homogeneity of the global DNRC distribution, for a fixed *k*, among all chromosomes of the genome, we used the phi coefficient,

2 $${\phi }_{k}=\sqrt{\frac{{\chi }_{k}^{2}}{n}},$$

where *n* is the total number of DNRC counts, as defined in (1), and ${\chi }_{k}^{2}$ is the Pearson’s chi-squared statistic,

3 $${\chi }_{k}^{2}=\sum _{w,j}\frac{{({O}_{w,j}-{E}_{w,j})}^{2}}{{E}_{w,j}},$$

where *O*_*w*,*j*_ is the observed frequency count of distances from *w* to *w*′ in chromosome *j*, and *E*_*w*,*j*_ is the expected frequency count under homogeneity, with $w\in {{\mathscr{A}}}^{k}$ and $j\in \{1,\ldots ,22,X,Y\}$.

The assumption of homogeneity of the distance distributions of the chromosomes allows us to discuss the statistical properties of the complete genome based on a sequence with all chromosomes concatenated.

### Residual analysis

From the perspective of molecular evolution, DNA sequences may reflect both the results of random mutation and of selective evolution. In order to highlight the contribution of selective evolution, one should subtract the random background from the simple counting result^19, 20^. To this purpose, the global DNRC distributions expected under the *k*-order Markov dependence, ${f}_{k}^{e}$, were obtained and the goodness-of-fit was evaluated by the *φ* measure (*φ* = 0 reveals a perfect fit between the distributions). To explore the differences between the empirical and the expected distributions, a residual analysis was carried out through the calculation of standardised residuals for a given distance *d*, are given by

4 $$r(d)=\frac{{f}_{k}(d)-{f}_{k}^{e}(d)}{\sigma },\,d > k,$$

where *n* is the total number of observed distances between symmetric pairs of length *k* and $\sigma =\sqrt{{f}_{k}^{e}(d)(1-\tfrac{{f}_{k}^{e}(d)}{n})}$ is the standard deviation of a binomial distribution. These standardised residuals are used to highlight the contribution of the selective evolution on the relative position of the symmetric word pairs.

We recall that, under *k*-order Markov dependence assumption, each standardised residual has an asymptotic standard normal distribution^21^.

The focus of this study is mainly in the short distances between symmetric word pairs, thus we fixed a maximal distance to 1000. The global Type I error was fixed to *α* = 5% and, for each distance comparison test, it was correct to 0.05 (1000 − *k*). So, absolute residuals greater than four are considered to be significant residuals.

Short distances between reversed complements may be related with the occurrence of cruciform structures, with maximum loop length of twenty nucleotides^6^. To identify a thresholding distance which may discriminate the overrepresented short distances from the underrepresented, we assumed that short distances up to the threshold are overrepresented and the others are underrepresented (this assumption makes sense under the hypothesis of enrichment of words able to form cruciform structures).

We determined the thresholding distance, *d*, as the distance that maximises the sum of the number of significant positive residues less than *d* and the number of significant negative residues greater than *d*. We defined a *discriminator function* as a sum of indicator functions (for example, ${{\mathbb{1}}}_{SP(i)}=1,$ if *r*(*d*) > 4)

5 $$R(d)=\sum _{i=k+1}^{d-1}{{\mathbb{1}}}_{SP(i)}+\sum _{i=d+1}^{1000}{{\mathbb{1}}}_{SN(i)},\,d > k,$$

where $SP(d)=\{d|r(d) > 4\}$ and $SN(d)=\{d|r(d) < -4\}$ and *r* is defined in Equation 4. The value *d* that maximises the discriminator function, *R*, was considered as the thresholding distance.

## Results and Discussion

The global DNRC distribution was determined for each of the 24 human chromosomes and each word length $(k=1,\ldots \mathrm{,\; 7})$. These distributions have heavy tails, strongly affecting the chi-square statistic. To avoid this problem, for each *k*, a cutoff distance was defined as the 99th percentile of the DNRCs observed in the complete genome (all chromosomes). Distances larger than this cutoff were lumped together into a residual class, in each distribution. Naturally, the DNRCs and hence the cutoff distances were found to increase with word length in the human genome, as would be expected even in a sequence of randomly generated nucleotides.

We measured the degree of homogeneity (*φ* effect sizes measure) between the human chromosomes, for the global DNRC distributions. According to the obtained *φ* values (*φ* < 0.04), we conclude that the homogeneity effect is weak. Thus, we consider that there is homogeneity between the global DNRC distributions of the several chromosomes. This chromosome homogeneity in the global DNRC distributions points to a general feature of the complete human genome, which may be due to genomic architecture constrains.

### Global DNRC distributions for the complete genome

The discrepancies between the global DNRC distribution in the human genome and in the *k*-order Markov process were measured by *φ* effect size measure. Although the misfit effect is not strong, it is nevertheless non-negligible. The *φ* values are always greater than 0.05 and the p-values smaller than 0.05.

**Figure 1:**
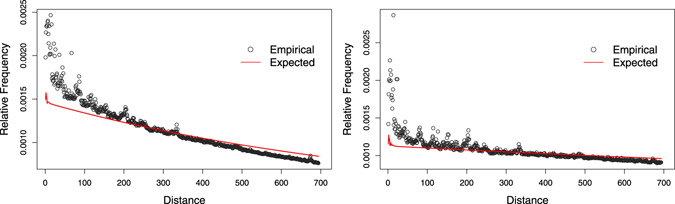
Empirical and expected global DNRC distributions, for the complete human genome, for *k* = 6 (left) and *k* = 7 (right). The expected distributions were obtained under the *k*-order Markov dependence assumption.

Figure 1 shows the global DNRC distributions of the human genome and the global DNRC distributions of the *k*-order Markov random sequence, for *k* = 6 and *k* = 7. The misfit between the human distance distributions and the corresponding *k*-order Markov process is clear. Analysing the residuals between the empirical distribution and the distribution of this random background, we observe a tendency of overrepresentation of short distances in the human genome, for all analysed values of *k*.

**Figure 2:**
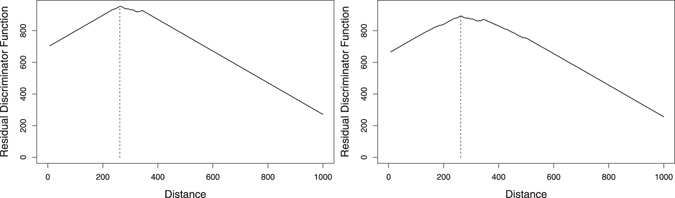
Residual discriminator function (*R*) for global DNRC distributions, relatively to the complete human genome, for *k* = 6 (left) and *k* = 7 (right). Both reach their maximum at distance 262.

Figure 2 presents the results of the residual discriminator function (the *R* profile), for the global DNRC distribution of the complete human genome (between the observed and the corresponding *k*-order Markov process), for *k* = 6 (left) and *k* = 7 (right). The discriminator functions increase for $d\mathop{ < }\limits_{ \tilde {}}260$, showing an evident favouring of short distances, and decrease for $d\mathop{ > }\limits_{ \tilde {}}350$. Both functions reach their maximum at distance 262. In fact, the human genome seems to favour the occurrence of shorter distances.

### Islands of favoured distances

**Figure 3:**
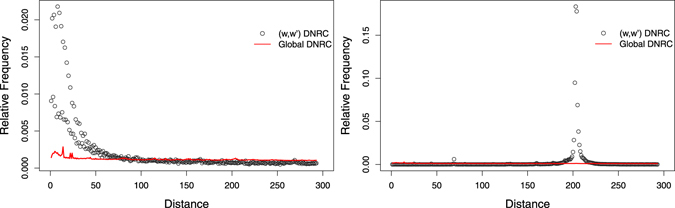
DNRC distribution, *f*_*w*,*w*′_, and global DNRC distribution, *f*_*k*=7_, for complete human genome. Overrepresentation of short distances in different ranges: *w* = *ATATATG* (left), *w* = *GGCTCAC* (right).

In this analysis, we computed all 4^*k*^ DNRC distributions, *f*_*w*,*w*′_. The plots of all the empirical DNRC distributions, for *k* = 6 and *k* = 7, are available in the Supplementary Material. Comparing each DNRC distribution with the global DNRC distribution, *f*_*k*_, it is possible to identify words which surpass the global behaviour observed for short distances (see, for example, Fig. 3).

We fixed [*k* + 1, *d*_*M*_] as the interval of interest, where *d*_*M*_ is the distance where *R* reaches the maximum value in the global distance distribution. We detected a subset of symmetric word pairs having DNRC distributions with an enrichment of distances in the interval of interest, when compared to the global DNRC distribution. Those distributions display a non-negligible misfit in relation to *f*_*k*_, for distances in [*k* + 1, *d*_*M*_]. However, not all words are significantly enriched (see the Supplementary Material).

Although some words may be visually identified as having enrichment of short distances, it is not always possible to perform meaningful statistical analysis, due to the small number of occurrences of the DNRC. Moreover, the runs of significant positive residuals (*r* > 4) are related with the ranges of overrepresented short distances.

The following procedure was developed to identify the DNRC distributions containing islands of favoured short distances, for a given *k*:

- We exclude the symmetric word pairs with occurrence frequency lower that 0.0001;
- We exclude the pairs (*w*, *w*′) such that *f*_*w*,*w*′_(*d*) = 0 for more than 5% of the distances *d* in [*k* + 1, 1000];
- The misfit between *f*_*w*,*w*′_ and *f*_*k*_ is evaluated by the phi coefficient. The symmetric word pairs with *φ* > 0.80 are considered to have a very strong effect size^22^. Symmetric pairs with phi coefficient below 0.8 are removed;
- For distances up to *d*_*M*_, the lengths of the longest run of significant positive residuals are considered. Symmetric word pairs with the longest positive run less than 25 are removed.

The four successive filters of the procedure above reduce the initial set of words to 17, for *k* = 6, and to 48, for *k* = 7. Note that other thresholds could have been used in the procedure, which would result in the selection of different subsets of words (see the Supplementary Material).

In order to classify the shape of the DNRC distribution of each symmetric word pair, a residual discriminator function *R* was obtained for each word pair, based on adjusted Pearson residuals, *r*_*a*_, computed from the contingency table of all words of length *k* and distances between (*k* + 1) and 1000, instead of the standardised residuals (eq. 4). The adjusted Pearson’s residuals are given by

6 $${r}_{a}(d)=\frac{{f}_{w,{w}^{^{\prime} }}(d)-{f}_{k}(d)}{\sqrt{{f}_{k}(d)}}\sqrt{n},$$

where *n* is the total number of DNRC counts for a given word length *k*, as defined in (1).

**Figure 4:**
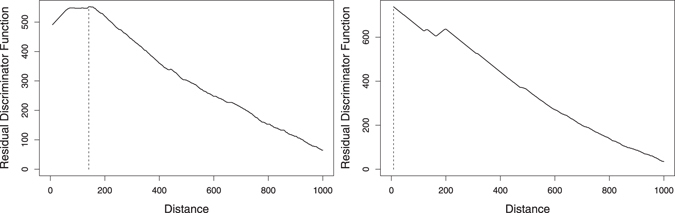
*R* profile for residuals between *f*_*w*,*w*′_ and *f*_*k*=7_, for the complete human genome. Different types of patterns: type *T*_1_ for *w* = *TATATAC* (left), and type *T*_2_ for *w* = *TCACGCC* (right).

The symmetric word pairs were classified in three different types, according to the *R* graphical profile:

*T*_1_ - A profile showing a marked initial increase, reaching its maximum, and stabilising or decreasing after it; see, for example, Fig. 4 (left);

*T*_2_ - A profile showing an initial decrease, comprising smooth or strong inverted peaks; see, for example, Fig. 4 (right).

*T*_3_ - Other profiles, not matching previous criteria.

The pairs of type *T*_1_ are characterised by high residual discriminator values (max(*R*) > 50), and their DNRC distributions show an enrichment for short distances (*d* < 100). See, for example, Fig. 3, left. Pairs of type *T*_2_ also have high residual discriminator values, but their DNRC distributions show an overrepresentation for distances *d* > 100, with localised bell-shaped peaks. See, for example, Fig. 3, right. All pairs of type *T*_3_ have irregular low-*R* profiles (max(*R*) ≤ 50).

Table 1 presents the subset of symmetric word pairs obtained by our procedure, for *k* = 7. The table also contains the maximum DNRC frequency and the corresponding distance, the max(*R*) values, the distribution type, and the distance peak location class. It was observed that type *T*_1_ is the largest group and is formed by *TA*-rich words. Most DNRC distributions of this type reach their maxima for *d* < 100 (C1). Curiously, it was reported that, in *E*. *coli*, cruciform formation is enhanced by *TA*-rich sequences and may correlate with transcriptionally-active promoters^23, 24^. Also, the cruciform-binding protein PARP-1 (Poly(ADP-ribose) polymerase-1), which is involved in DNA recombination and repair, was shown to interact with promoter-localised cruciforms^25^, and promoters are frequently enriched with TA elements^26^. Thus, the overrepresentation of short distances of *TA*-rich symmetric word pairs, detected by the procedure that we propose, may point to the occurrence of hairpin/cruciform structures.

**Table 1:** Words of length seven with DNRC overrepresentation of short distances, identified by our procedure, with indication of DNRC distribution maximum and its argument value, discriminator *R* function maximum, group type (*T*1 and *T*2) and class of peak distance.

*w*	max(*f*_*w*,*w*′_)	*arg* max(*f*_*w*,*w*′_)	max(*R*)	Type	Class
ATATATA	0.04	9	932	*T*_1_	C1
GTGTATA	0.05	9	57	*T*_1_	C1
TATATAT	0.03	10	881	*T*_1_	C1
TATATAC	0.03	15	552	*T*_1_	C1
GTATATA	0.05	9	76	*T*_1_	C1
ATATATG	0.02	15	453	*T*_1_	C1
TATATGT	0.02	13	301	*T*_1_	C1
TATATAA	0.03	13	566	*T*_1_	C1
ATATACA	0.03	13	248	*T*_1_	C1
TGTGTAT	0.03	9	60	*T*_1_	C1
TTATATA	0.03	9	83	*T*_1_	C1
TATATTA	0.04	11	109	*T*_1_	C1
TATACAC	0.02	294	62	*T*_1_	C4
TATAATA	0.04	9	96	*T*_1_	C1
CATATAT	0.03	9	68	*T*_1_	C1
TGTATAT	0.03	9	118	*T*_1_	C1
TATACAT	0.03	9	92	*T*_1_	C1
AATATAT	0.02	9	109	*T*_1_	C1
ATATAAT	0.03	11	134	*T*_1_	C1
ATATTAT	0.04	9	90	*T*_1_	C1
ATTTTAT	0.03	107	271	*T*_1_	C2
ATACATA	0.03	9	77	*T*_1_	C1
TATGTAT	0.02	9	87	*T*_1_	C1
TATGTGT	0.02	15	56	*T*_1_	C1
ATATGTA	0.02	11	114	*T*_1_	C1
ATGTATA	0.02	15	71	*T*_1_	C1
ATATATT	0.02	13	486	*T*_1_	C1
ACATATA	0.02	11	71	*T*_1_	C1
TATTATA	0.02	9	63	*T*_1_	C1
TACATAT	0.02	13	75	*T*_1_	C1
ATACACA	0.01	15	59	*T*_1_	C1
TAATATA	0.02	13	73	*T*_1_	C1
TCACGCC	0.33	179	738	*T*_2_	C3
GTTCAAG	0.27	122	913	*T*_2_	C3
GGCTCAC	0.18	210	935	*T*_2_	C4
TGGCTCA	0.14	213	911	*T*_2_	C4
TTGAGAC	0.14	199	857	*T*_2_	C3
CAGTGGC	0.12	230	820	*T*_2_	C4
GCAGTGG	0.10	232	700	*T*_2_	C4
TTTGAGA	0.12	201	776	*T*_2_	C3
GTGCAGT	0.08	236	347	*T*_2_	C4
ATCATGG	0.04	148	116	*T*_2_	C3
ATCTCAT	0.04	121	54	*T*_2_	C3
CCTGGGC	0.03	115	91	*T*_2_	C3

Distance peak classes: *C*_1_ (*d* < 100), *C*_2_ (*d* ≈ 100), *C*_3_ (100 < *d* < 200) and *C*_4_ (*d* > 200).

The proposed procedure also identifies the *T*_2_ group. DNRC distributions in this group have localised bell-shaped peaks for *d* > 100, forming marked islands of favoured distances. The occurrence of peaks in the short distance region of the DNRC distribution could signal the formation of hairpin/cruciform structures. However, DNRC distribution peaks for *d* > 100 could be associated to other structural or functional DNA functions.

### Single over-favoured distance

Apart from the words that have clear islands of favoured distances, in the complete list of words of length six and seven (see Supplementary Material) several words can be observed with a single distance very highlighted, due to its high frequency. In order to perform an automatic selection of this kind of words, we defined the procedure:

- We start with the complete set of symmetric words of a fixed length *k*;
- We exclude the symmetric word pairs with occurrence frequency lower that 0.0001;
- We exclude the pairs (*w*, *w*′) such that *f*_*w*,*w*′_(*d*) = 0 for more than 5% of the distances *d* in [*k* + 1, 1000];
- The remaining words are sorted by the maximum frequency, max(*f*_*w*,*w*′_), of the distances under analysis $d=k+1,\ldots ,1000$);

The first words obtained by these criteria identify words with a single over-favoured distance. To the purpose of classifying the words with relation to single favoured distance, we defined three subsets: peak in distances *d* ≤ 30, peak in distances 30 < *d* ≤ 200 and peak in distances *d* > 200. Table 2 shows the first five words obtained by the previous procedure, for each peak interval type. Taking into account the expected decrease of the distribution, the peak in distances *d* > 200 is an obvious unexpected behaviour. It is noteworthy that for some words a single distance accounts for about 70% of occurrences in a total of (1000 − *k*) distances.

**Table 2:** Words with highest *f*_*w*,*w*′_ maximum, with indication of maximising distance, for word length 7, organised by peak type: *d* ≤ 30, 30 < *d* ≤ 200 and *d* > 200.

Peak type	*w*	max(*f*_*w*,*w*′_)	*arg* max(*f*_*w*,*w*′_)
*d* ≤ 30	CATTAGG	0.78	14
	TGCAGTG	0.77	21
	CATGTCC	0.71	14
	TCAACTC	0.71	10
	TTCAACT	0.66	12
30 < *d* ≤ 200	TAGCTGG	0.67	31
	GTTGAAC	0.60	157
	TGTTCTC	0.46	31
	CCACAAT	0.45	133
	GAGTTGA	0.43	161
*d* > 200	CCATGCT	0.28	251
	TCCCCAT	0.25	292
	GAATTCT	0.22	339
	TGAATGG	0.22	344
	ATGGGAT	0.21	490

**Figure 5:**
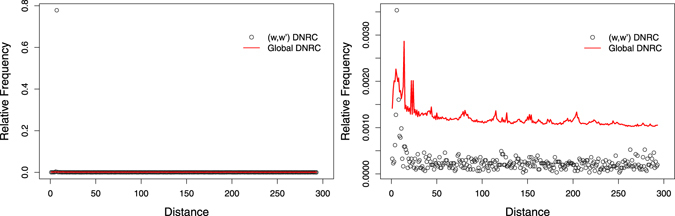
DNRC distribution *f*_*w*,*w*′_ for *w* = *CATTAGG*, and global DNRC distribution, *f*_*k*=7_, for the complete human genome. Very strong enrichment for distance 14: *f*_*w*,*w*′_(14) = 0.778 (left). The right plot is a zoom of *y* axis.

**Figure 6:**
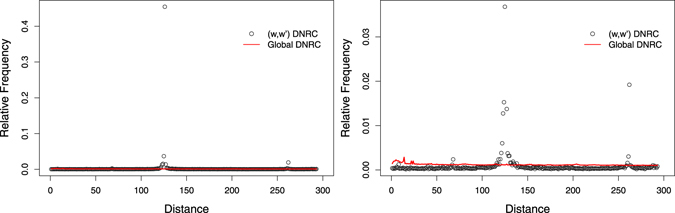
DNRC distribution *f*_*w*,*w*′_ for *w* = *CCACAAT*, and global DNRC distribution, *f*_*k*=7_, for the complete human genome. Very strong enrichment of distance 133: *f*_*w*,*w*′_(133) = 0.455 (left). The right plot is a zoom of *y* axis.

Figure 5 presents the DNRC distribution of *CATTAGG* (first word in Table 2). This symmetric word pair shows a single over-favoured distance *d* = 14 (${f}_{w,{w}^{^{\prime} }}\mathrm{(14)}\approx 0.8$). In a *y*-axis zoom (right), a local island of favoured distances is observed. However, in general, these frequencies do not surpass the global distance distribution behaviour. Figure 6 shows another example of a symmetric word pair with a single over-favoured distance at *d* = 133.

In the absence of obvious biological motivation for the occurrence of these single over-favoured distances, we conducted further analyses for some word pairs that have these features. To address the possibility that the reported behaviour may result from a sequencing procedure artefact, we studied the pair (*CCACAAT*, *ATTGTGG*) in detail. Using three independently sequenced and assembled genomes (Celera, HuRef, GRCh38.p2), we computed the distance distributions and found a similar peak in each (see Table 3, displaying the frequencies around distance 133), ruling out the hypothesis that the observed distance peaks are sequencing or assembly artefacts.

**Table 3:** DNRC partial distribution of *CCACAAT*, around distance 133, for three distinct human genome assemblies (Celera, HuRef, GRCh38.p2).

Distance	Celera	HuRef	GRCh38.p2
125	76	81	81
126	35	40	43
127	78	82	83
128	117	125	131
129	196	205	206
130	402	426	437
131	469	512	524
132	1103	1235	1263
133	12962	14938	15599
134	528	472	472
135	131	126	129
136	98	109	108
137	98	101	109
138	46	56	56
139	52	51	55

We further analysed the sequences comprised between *CCACAAT* and *ATTGTGG*. Taking into account the sequence direction, the distance 133 was only enriched for *CCACAAT* to *ATTGTGG* (15599 occurrences) but not for *ATTGTGG* to *CCACAAT* (11 occurrences, which is in the expected range). The sequence logo (not shown) for the 15599 sequences of the GRCh38.p2 human genome, obtained using WebLogo 3.4^27^, shows a significant degree of conservation, suggesting that these sequences may be part of repetitive DNA segments. Using the genomic coordinates for the *CCACAAT* words which are at distance 133 from *ATTGTGG* words, we searched the RepeatMasker annotations available from the UCSC Table Browser. From the 15599 occurrences, 15586 locate within Long INterspersed Elements (LINEs), specifically from the L1 retrotransposon family. L1 are active transposable elements that also mobilise non-autonomous elements, such as Alu sequences, thus shaping the genome landscape and variation, with implications in evolution and disease^28, 29^.

#### Masked Sequences

To reduce bias from known repetitive sequences in the original genome assembly, we also analysed a pre-masked version of the genome (as reported by RepeatMasker and Tandem Repeats Finder). Masked sequences exclude major known classes of repeats^30^, such as long and short interspersed nuclear elements (LINEs and SINEs), long terminal repeat elements (LTRs), Satellite repeats or Simple repeats (micro-satellites).

As expected, masking eliminates distance peaks in several DNRC distributions. For instance, the DNRC distribution of *w* = *CCACAAT* (Fig. 6) loses the strong peak observed for the complete genome, because the enrichment of distance 133 is due to LINEs repeats. However, the peaks are preserved in several other distributions.

To select words with single over-favoured distances, in these masked sequences, we applied the procedure described in the previous section. As before, results were classified in three subsets.

The highest-ranking words in the *d* ≤ 30 group are *TA*-rich words. Also, we observed that the shape of the DNRC distributions for these words remain unchanged by the masking of repeats. These distributions preserve their characteristic islands of enriched short distances. The distributions of highest-ranking words in the other two groups do not show islands of favouring distances. They display just one or a few strong peaks in the repeat-masked genome.

Globally, the distance peak of the DNRC distributions in the repeat-masked genome correspond to a local maximum in the original genome (80% of the distributions).

**Figure 7:**
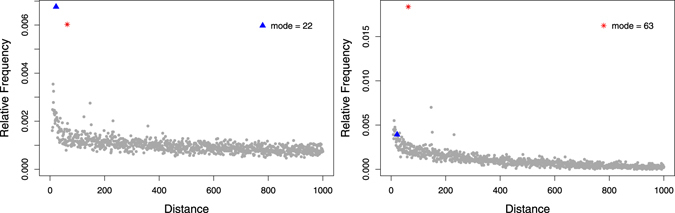
DNRC distribution *f*_*w*,*w*′_ for *w* = *TATGAAT* in the complete genome (left) and in the repeat-masked genome (right). The triangle symbol identifies the mode in the complete genome (*d* = 22) and the asterisk symbol is the mode in the masked genome (*d* = 63).

Table 4 shows the first five words obtained for each subset, for *k* = 7. The words reported in Table 4 do not show up in the top-5 list of the complete genome sequence (Table 2). Nevertheless, the peaks detected in their distributions are also local maxima, or even global, in the corresponding non-masked distribution. As an example, Fig. 7 shows the DNRC distribution of *TATGAAT* in the complete genome (left) and in the repeat-masked genome (right). Distance 63 is the distribution mode (peak) in the repeat-masked genome, and also a local maximum in the distribution extracted from the complete genome.

**Table 4:** Words with highest *f*_*w*,*w*′_ maximum, with indication of the maximising distance, in masked sequences, organised by peak type: *d* ≤ 30, 30 < *d* ≤ 200 and *d* > 200.

Peak type	*w*	max(*f*_*w*,*w*′_)	*arg* max(*f*_*w*,*w*′_)	chr
*d* ≤ 30	TGTGTAT	0.033	9	several
	TATATAT	0.031	13	several
	AATATAT	0.027	9	several
	TGTGTGC	0.027	9	several
	ATATACA	0.027	13	several
30 < *d* ≤ 200	GGGCCCA	0.033	101	chr13
	CAGGCTC	0.023	31	chr1
	AAGCTTT	0.020	83	chr19
	TATGAAT	0.018	63	chr19
	GCCACAG	0.013	115	chr1
*d* > 200	GTTTTCC	0.010	425	chr1
	TGAAATC	0.010	555	chr1
	GGCTCAG	0.009	401	chr1
	TGAGAGA	0.009	502	chr1
	TTTTGTC	0.009	256	chr1

The peaks of DNRC distributions of words in Table 4 were analysed in order to assess the existence of biological features. The peak distances in the *d* ≤ 30 subset arise from the overall contribution of several chromosomes. For the words in the other subsets, there is clearly a chromosome that is the main contributor to the single distance peak (see Table 4). Annotations within genomic coordinates for the words listed in Table 4 were retrieved from UCSC GENCODE v24 (https://genome.ucsc.edu/cgi-bin/hgTables) and the resulting gene lists were analysed with the functional annotation tool in DAVID^31, 32^. Overall, word pairs with peaks at distances *d* > 30 are enriched in genes with several and well-defined protein domains, namely, DNA-binding Zinc-finger proteins and members from the neuroblastoma breakpoint family (NBPF). These are duplicated genes with extreme copy number expansion that are associated with brain development and pathology, and are located in a human-specific pericentric inversion in chromosome 1^33, 34^. Word pairs with distance peaks at *d* ≤ 30 are scattered throughout the genome, and show enrichment in genes associated with the membrane, which also display a conserved protein topology. As in the *T*_1_ group of the complete genome, the words of this subset are *TA*-rich which may be associated with cruciforme structure occurrence.

## Conclusions

We developed new procedures to describe some characteristics of genomic words. In particular, the relative position and distance between reverse complemented word pairs was addressed, using the notion of distance to the nearest reversed complement (DNRC). Under this framework, we studied the DNRC distribution of each word in comparison with the global DNRC distribution and verified the homogeneity of the global DNRC distribution across human chromosomes, for word sizes 1 ≤ *k* ≤ 7.

Using these novel procedures for genomic word detection, we were able to find words with unexpected features in the DNRC distribution, which could not be detected by word frequency procedures alone. The detection of pairs of symmetric words that occur very often at a fixed distance (e.g., the pair (*CCACAAT*, *ATTGTGG*) at distance 133) suggests structural characteristics of the DNA. Some of these are already known but some others may be new.

We explored the global DNRC distributions of words of lengths *k* = 6 and *k* = 7 in the human genome, comparing them with the expected distributions obtained under *k*-order Markov dependence. A lack of fit was globally detected. The global DNRC distributions show a strong overrepresention of distances up to 350, a feature that may be associated with the occurrence of cruciform structures.

The DNRC distributions of some word pairs display significantly overrepresented distances. In the complete genome, those distributions fall into one of several distinct patterns: distributions with islands of favoured distances *d* < 100 (typically *TA*-enriched words); distributions with islands of favoured distances between 50 and 350; distributions with a single overrepresented distance. In the masked genome version, distributions with islands of favoured distances for *d* ≤ 30 (typically *TA*-enriched words) and distributions with single over-favoured distance for *d* > 30, were observed. Some of these peaks are present in both complete and masked genomes, thus they are not related to the major known classes of repeats.

DNA structures such as stem-loops and cruciforms are formed at sites that contain reversed complementary words. For this reason, their study naturally leads to the study of the symmetry properties of the sequences, and in particular to the study of the distribution of distances between nearest reversed complements. We performed an exhaustive study of these distance distributions and identified words that are strong candidates to the formation of cruciform structures in human DNA. We are convinced that the new procedures defined and proposed in this work are relevant for a better understanding of the structure of DNA.

## Electronic supplementary material

Supplementary information
