# Supplementary material for: DNA word analysis based on the distribution of the distances between symmetric words
Source: Sci Rep. 2017 Apr 7;7:728. doi: 10.1038/s41598-017-00646-2 (PMC5428789; doi:10.1038/s41598-017-00646-2)
Supplement: Supplementary file 1 — Supplementary information [file 41598_2017_646_MOESM1_ESM.pdf]

DNA word analysis based on the distribution of the distances between symmetric words

Ana H. M. P. Tavares, Armando J. Pinho, Raquel M. Silva, João M. O. S. Rodrigues, Carlos A. C. Bastos, Paulo J.S.G. Ferreira, and Vera Afreixo

Supplementary Material

Supplementary material is available online at

http://sweet.ua.pt/vera/DNRC%20-Supplementary%20material.html

It includes a zip file with the data-sets of all used features of empirical DNRC distribution for the complete genome, for all words of length six and seven, as also for all words of length seven in the repeat-masked genome.

To use this supplementary material, the reader should download the zip file and open the included Excel file. To see the distribution and R plot of word w, the reader should select the link with the word name in the corresponding column. To select words according to our criteria (or another), the reader should activate the Excel filters of the seven DNRC features, namely: (A) - proportion of distances (d) with fw;w0 (d) = 0; (B) - frequency of the symmetric words pair in the genome; (C) - j between fw;w0 and the global distribution; (D) - longest run of significant positive residuals between fw;w0 and the global distribution; (E) - maximum residual discriminator value (max(R)) of fw;w0 ; (F) - distance with maximal frequency; (G) - maximum frequency of DNRC distribution.
